# Therapeutic Potential of Antimicrobial Peptides in Polymicrobial Biofilm-Associated Infections

**DOI:** 10.3390/ijms22020482

**Published:** 2021-01-06

**Authors:** Giovanna Batoni, Giuseppantonio Maisetta, Semih Esin

**Affiliations:** Department of Translational Research and New Technologies in Medicine and Surgery, University of Pisa, 56123 Pisa, Italy; giuseppantonio.maisetta@dps.unipi.it (G.M.); semih.esin@med.unipi.it (S.E.)

**Keywords:** antimicrobial peptides, host defense peptides, polymicrobial infections, biofilms, mixed infections, wound infections, lung infections, *Pseudomonas aeruginosa*, *Staphylococcus aureus*

## Abstract

It is widely recognized that many chronic infections of the human body have a polymicrobial etiology. These include diabetic foot ulcer infections, lung infections in cystic fibrosis patients, periodontitis, otitis, urinary tract infections and even a proportion of systemic infections. The treatment of mixed infections poses serious challenges in the clinic. First, polymicrobial communities of microorganisms often organize themselves as biofilms that are notoriously recalcitrant to antimicrobial therapy and clearance by the host immune system. Secondly, a plethora of interactions among community members may affect the expression of virulence factors and the susceptibility to antimicrobials of individual species in the community. Therefore, new strategies able to target multiple pathogens in mixed populations need to be urgently developed and evaluated. In this regard, antimicrobial or host defense peptides (AMPs) deserve particular attention as they are endowed with many favorable features that may serve to this end. The aim of the present review is to offer a comprehensive and updated overview of studies addressing the therapeutic potential of AMPs in mixed infections, highlighting the opportunities offered by this class of antimicrobials in the fight against polymicrobial infections, but also the limits that may arise in their use for this type of application.

## 1. Introduction

Over the last few decades, the importance of studying microbes as part of mixed-species communities rather than in isolation has become increasingly recognized [1]. Many human infections are in fact polymicrobial including oral infections, infected surgical wounds or diabetic foot ulcers, otitis media, urinary tract infections and lung infections in cystic fibrosis (CF) patients [1] (Table 1).

Several types of biological interactions can be established among members of a community, ranging from parasitism (one organism benefits at the cost of another) to commensalism (one organism benefits with no cost for another) or to mutualism (a type of relationship whereby both organisms benefit) [20]. Numerous studies have highlighted that microbial interaction within mixed infections may accelerate and worsen disease progression [21,22,23,24], but examples of antagonistic interactions that protect the host from disease also exist [25].

**Figure 1:**
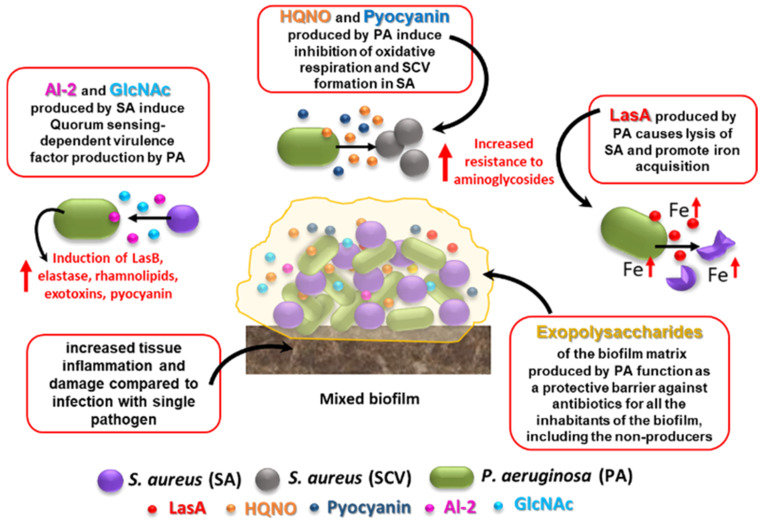
Effects of *S. aureus–P. aeruginosa* mixed infection on resistance and virulence as compared to single-species biofilms. Some of the best characterized interactions that occur between the two species in mixed biofilms are shown. See text for details. HQNO: 4-Hydroxy-2-Heptylquinoline N-Oxide; SCV: small colony variant; AI-2: autoinducer 2; GlcNAc: N-acetyl glucosamine, a component of bacterial peptidoglycan.

The treatment of polymicrobial infections adds additional therapeutic challenges as compared to their monomicrobial counterparts, as the efficacy of antibiotics or other antimicrobial agents may greatly differ when they are directed against single microbial species or towards communities composed of different combinations of microbes [26,27,28]. In this regard, one of the most studied examples is the interaction between *Staphylococcus aureus* and *Pseudomonas aeruginosa*, two bacterial species that often cohabit in chronically infected wounds or in the lungs of CF patients [24] (Figure 1).

For instance, it has been reported that *P. aeruginosa* exoproducts markedly decrease the sensitivity of *S. aureus* biofilms and planktonic populations to vancomycin, a frontline antibiotic used to treat methicillin-resistant *S. aureus* in CF patients [28]. Other reports have demonstrated that the *P. aeruginosa* exoproduct 4-hydroxy-2-heptylquinoline-N-oxide (HQNO) protects *S. aureus* from killing by commonly used aminoglycoside antibiotics such as tobramycin [29]. The identified mechanism was the ability of HQNO to induce the formation of small-colony variants (SCVs), slow-growing phenotypes of *S. aureus* exhibiting atypical colony morphology and exceptionally high and stable resistance to aminoglycosides and antifolate agents [30]. On the other hand, *P. aeruginosa* senses the presence of N-acetyl glucosamine (GlcNAc), a component of the cell wall peptidoglycan released by *S. aureus* during growth, commonly found in the CF lung [24]. GlcNAc enhances the *Pseudomonas* quinolone signal (PQS), which controls the production of a number of extracellular virulence factors involved in inflammation and tissue damage (e.g., pyocyanin, elastase, rhamnolipids and HQNO). PQS is one of the three quorum sensing (QS) systems present in *P. aeruginosa*; it uses HQNO as the main effector molecule, and is regulated by the other two *P. aeruginosa* QS systems, namely LasR (positive regulation) and RhIR (negative regulation). A similar virulence factor-inducing effect has been described for Autoinducer-2 (AI-2), a small diffusible QS molecule produced by several Gram-positive bacteria, including *S. aureus* [31] (Figure 1). As part of a competitive relationship with *S. aureus*, *P. aeruginosa* produces the endopeptidase LasA, a staphylolysin responsible for *S. aureus* lysis [24]. As the presence of *S. aureus* causes a down-regulation of the *P. aeruginosa* iron-regulated gene, it has been proposed that lysed *S. aureus* cells may represent for *P. aeruginosa* a source of iron in in vivo low-iron environments [32].

A major player in antibiotic tolerance and virulence during polymicrobial infections is biofilm formation [33]. Within a biofilm, an abundant extracellular polymeric substance (EPS) protects all microbial cells (including the non-producers) from a variety of harmful stimuli, including antibiotics and host defense factors (Figure 1). Adam et al. reported a striking example of how EPS alters antibiotic susceptibility in mixed infections [34]. They demonstrated that while an EPS-nonproducing mutant strain of *S. epidermidis* is normally highly sensitive to vancomycin, it is protected from the same antibiotic when grown in co-cultures with *C. albicans* [34]. On the other hand, the abundant EPS produced by the wild-type strain of *S. epidermidis* (RP62A) can inhibit fluconazole penetration in mixed fungi-bacteria biofilms, protecting *C. albicans* from the action of the antifungal drug [34]. Recently, an interesting mechanism by which *C. albicans* may promote multidrug tolerance in *S. aureus* was proposed [35]. *S. aureus* grown in dual cultures with *C. albicans* was found to display decreased intracellular ATP levels and lower membrane potential as compared to cultures lacking *C. albicans*. *C. albicans*-mediated nutrient deprivation was shown to cause decreased metabolic activity in *S. aureus*, inducing the formation of persisters, dormant cells highly tolerant to antibiotic treatment. Members of a polymicrobial biofilm may also produce antibiotic-modifying enzymes (e.g., β-lactamases) of which not only the producing species, but also the co-infecting species may benefit. Interspecies horizontal gene transfer is another mechanism that might facilitate the acquisition of antibiotic-resistant genes within a polymicrobial biofilm [33].

Despite the numerous examples correlating interspecies interactions in mixed infections with variations in pathogenicity and the antibiotic susceptibility of individual organisms, antibiotic therapies are often directed towards the most relevant pathogen, disregarding the consequences that the presence of other bacterial species may have in the pathogenicity and the response to antimicrobial therapy [36]. Therefore, new strategies targeting multiple pathogens in mixed populations and considering the multifaceted interactions that are established in the community need to be evaluated.

The interest in the use of antimicrobial or host defense peptides (AMPs) as antibiofilm agents has rapidly grown over the last few decades [37]. Many AMPs have shown activity in killing cells in biofilms, interfering with EPS production and stability, inhibiting QS-dependent biofilm formation, or preventing microbial adhesion when used to coat medical implants [37,38]. A manually curated database of AMPs specifically assayed against microbial biofilms (http://www.baamps.it/) was issued for the first time in 2015 [39] and has stimulated the development of several computational approaches to accurately predict anti-biofilm peptides [40,41,42]. Such approaches have revealed a prevalence of positively charged and aromatic residues, and the selective presence of some dipeptides and sequence motifs in biofilm inhibiting peptides (BIP) as compared to non-BIP, aiding the choice of potential AMP-candidates to direct toward preclinical development.

**Figure 2:**
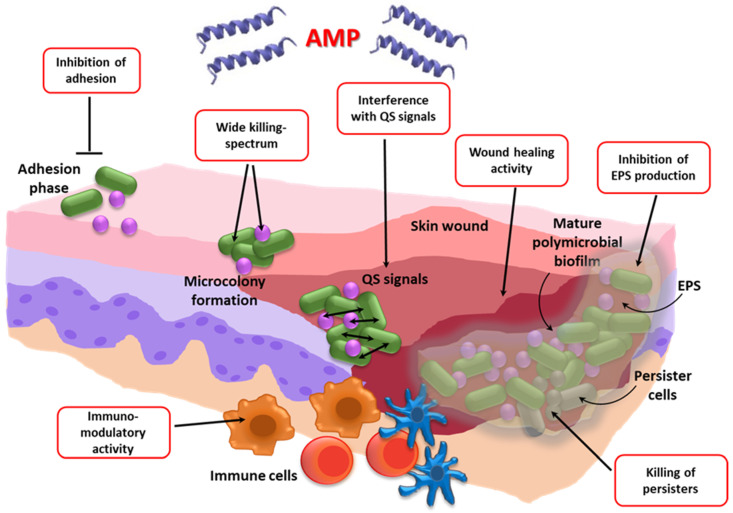
Formation stages of a polymicrobial biofilm in a skin wound taken as an example of mixed biofilm-associated infections. The red boxes show the multiple mechanisms of action proposed for the antibiofilm activity of AMPs. AMP: Antimicrobial peptide; QS: Quorum sensing; EPS: Extracellular polymeric substances.

Despite the keen interest in AMPs as antibiofilm agents, their possible use against biofilm-associated polymicrobial infections is a relatively poorly investigated research area, but it has the potential to offer innovative and effective solutions for the treatment of co-infections (Table 2, Figure 2).

Firstly, AMPs often exert a wide spectrum of activity directed not only against Gram-positive and Gram-negative bacteria, but also towards fungi, viruses, and protozoa. This factor may represent an advantage over conventional antibiotics, especially in multidrug-resistant (MDR) infections, where the last-resort antibiotics, often narrow-spectrum, must be used (e.g., colistin against MDR *P. aeruginosa* or vancomycin against methicillin-resistant *S. aureus*). Secondly, unlike most antibiotics that target active cell processes, AMPs may act also against persisters that populate biofilms in high frequencies, and are regarded as major contributors to the relapsing nature of many biofilm-associated infections [43,44]. In this regard, we have recently demonstrated that AMPs of different origins and structures kill persister cells of both *P. aeruginosa* and *S. aureus* [43]. Each peptide exerted a broad-spectrum killing effect after short incubation times (3 h) and at concentrations similar to or even much lower than those of licensed membrane-targeting antibiotics, such as colistin and daptomycin. Thirdly, AMPs are often multifunctional molecules endowed with wound-healing and/or anti-inflammatory activities [45,46]. Last but not least, AMPs are versatile and prone to modifications that may optimize their antimicrobial and/or anti-inflammatory properties, or that allow for targeting them towards individual species within a mixed community [47,48]. Despite these favorable properties, obstacles to AMP-use in mixed infections may arise (Table 2), including the insurgence of community-based AMP-resistance mechanism, warranting further investigations to fully explore their potential in such kinds of infections.

The aim of the present review is to offer a comprehensive and updated overview of the studies addressing the therapeutic potential of AMPs in mixed infections (Table 3), highlighting the opportunities offered by this class of antimicrobials in the fight against polymicrobial infections, but also the limits that may arise in their use for this type of application. Selected examples of the AMPs tested against polymicrobial infections are discussed in the following paragraphs.

## 2. Bacteria–Bacteria Mixed Infections

### 2.1. Wound Infections

Due to the general aging of the world population, there is an increasing number of patients suffering from chronic diseases such as diabetes, cardiovascular diseases, cancer, and immunosuppression. Many of these conditions are causes of chronic wounds that include diabetic foot ulcers (DFU), venous leg ulcers, or pressure ulcers, and are estimated to be experienced by 1–2% of the population of developed countries during their lifetime [68]. The bacterial colonization of chronic wounds may lead to biofilm formation, which elicits local and systemic inflammation and negatively affects the healing process [69]. Chronic wounds are typically colonized by more than one bacterial species [5], with *S. aureus* and *P. aeruginosa* among the most common ones [6].

#### 2.1.1. Conventional Therapy

Despite the high prevalence and the massive financial burden placed by chronic wounds on the healthcare system, innovations in clinical management and wound care have been scarce in the past century [70]. To prevent the bacterial infection of skin wounds, widely used antimicrobials such as iodine, silver, zinc oxide, and polyhexamethylene have a broad spectrum of antibacterial activity, but they are endowed with certain levels of cytotoxicity [71]. Routine therapeutic strategies (e.g., systemic use of antibiotics, operative debridement) require a long course of treatment, are expensive, and fail to produce satisfactory results [72]. Therefore, the development of new and more effective antimicrobials for clinical application is highly desired.

#### 2.1.2. AMP-Based Therapy

AMPs are part of the innate skin defense mechanisms providing a first-line barrier to microbial insult [73]. Skin AMPs include β-defensins (BD), cathelicidins (human hCAP18/LL37), RNase 7, chemerin, and secretory leukocyte protease inhibitor (SLPI) [73]. For example, hCAP18/LL37, one of the best-characterized peptides in skin defense, is upregulated in the epidermis as a result of skin injury and infection, while mice deficient in the murine homolog of hCAP18/LL37 (CRAMP) are more susceptible to serious cutaneous streptococcal infections [74], highlighting the importance of AMPs in skin protection against bacteria.

AMPs hold promise as new therapeutic agents for infected wounds due to their broad activity spectrum, antibiofilm potential, immunomodulatory action, angiogenetic and wound-healing properties, and their ability to stimulate cell proliferation and migration [45,75,76,77]. However, only a relatively small number of AMPs have been tested as a new therapeutic strategy to prevent or treat polymicrobially infected wounds (Table 1). For instance, Chung and coworkers designed and synthetized a new short AMP (named DRGN-1), a derivative of the VK25 peptide found in the plasma of the Komodo dragon (*Varanus komodoensis*), a large species of lizard found on the Indonesian island of Komodo [49]. They demonstrated that the peptide significantly inhibits single species and mixed-species biofilms of *P. aeruginosa* and *S. aureus* in vitro at 24 h, as evaluated by the crystal violet staining of biofilms and confocal microscopy. The peptide was also tested in a mouse wound infection model. To this aim, full-thickness, 6 mm diameter round wounds were overlaid with a mixed biofilm of *P. aeruginosa* and *S. aureus* grown on agar for two days, and the kinetics of wound closure and the bacterial load were evaluated after peptide treatment. The results obtained demonstrated the ability of DRGN-1 to accelerate wound closure and reduce the bacterial count of both species. The efficacy of DRGN-1 to stimulate keratinocyte migration in a scratch-wound closure assay was also demonstrated, further stressing the potentiality of the peptide in the therapy of infected wounds.

The antimicrobial efficacy of individual AMPs can be greatly enhanced by combining them with other AMPs or with other antimicrobial agents [78]. Combination therapies have the undisputed advantage of being able to reduce the insurgence of resistance as well as the active concentrations of the combined drugs, with consequent attenuation of cytotoxicity and possible side effects. Gomes et al. recently assessed the combination of two AMPs, pexiganan and nisin, for their ability to control polymicrobial diabetic foot infections [50]. When tested against planktonic and biofilm cells of *S. aureus*, the dual AMP displayed an increased activity compared to pexiganan used alone, but this was not the case for *P. aeruginosa* monocultures or dual species cultures. It was suggested that the scarce effect elicited by adding nisine to pexiganan to target *P. aeruginosa* was due to nisin’s mode of action, which relies on its ability to bind lipid II with the consequent inhibition of cell wall biosynthesis [79]. As lipid II is located in the cytoplasmic membrane, the presence of an outer membrane in Gram-negative bacteria may hamper the peptide’s ability to reach its target, with reduced antimicrobial efficacy. A DFU collagen three-dimensional (3D) model was used to evaluate further the efficacy of the locally delivered dual AMP, incorporated in a guar gum biogel. When *S. aureus* and *P. aeruginosa* were inoculated as a dual species inoculum into the model, the strong antibacterial activity of the dual AMP biogel was observed against *S. aureus*, resulting in bacterial eradication from three different areas of the collagen scaffold. In contrast, the activity of the dual AMP biogel was null or scarce against *P. aeruginosa*, which was detected, instead, in all the areas of the model. These results highlight that *P. aeruginosa* might be a bacterial species particularly difficult to target with both conventional antibiotics and AMPs. In addition, the data obtained suggest that the mechanisms of action of the peptide(s) employed should be taken into consideration to target all the species of a mixed community with equal efficiency. Jorge and coworkers explored another AMP-based combination strategy, testing colistin sulfate salt (CST) combined with the AMPs temporin A (TEMP-A), citropin 1.1 (CIT-1.1) or tachyplesin I linear analogue (TP-I-L) against single and dual species biofilms of the two major wound pathogens *P. aeruginosa* and *S. aureus* [51]. They demonstrated synergistic/additive or indifferent activity against 24-h-old dual species biofilms, depending on the antimicrobial combination and strain tested (reference strains, or MDR clinical isolates). Although in mixed biofilms the initial bacterial number was the same for the two species, at 24 h the biofilms were predominantly composed of *P. aeruginosa*, suggesting the establishment of competitive interactions between the two species during the incubation period. The AMP concentration required to target the dual species biofilms was overall higher than that required to treat mono-species biofilms, with some of the combinations demonstrating a high level of cytotoxicity against mammalian cells [51].

Despite their potential, the delivery of AMPs for topical applications represents a challenge as they are susceptible to degradation by bacterial and host proteases and/or sequestration by molecules present in the wound environment (e.g., serum proteins). Therefore, the development of appropriate delivery systems to increase peptide stability, and reduce peptide-mediated toxic effects, while ensuring a sustained and long-term peptide release, is considered critical to maximize the antimicrobial and wound healing effects [80,81]. A de novo designed cationic, amphiphilic peptide (ASP-1), formulated within a hydrophilic polyurethane (PU)-based dressing, was evaluated in vitro against MDR wound pathogens [52]. A polymicrobial poloxamer biofilm model was used for this aim. In the model, a 30% poloxamer 407 cold solution is mixed with a bacterial suspension and layered on a glass slide where the poloxamer forms a gel when it reaches room temperature, simulating the biofilm conditions. The polymicrobial biofilm, consisting of four species (*S. aureus* MRSA 6313, *A. baumannii* 6043, *K. pneumonia* 6066, and *P. aeruginosa* 6162 obtained from the clinical isolate collection at Trideum Biosciences, Frederick, MD, USA), was covered with the ASP-1-loaded PU dressing and incubated at 37 °C for 24 h. Total bacterial count was then assessed and compared to that of a solution of ASP-1, the gauze control, the placebo dressing and a commercial silver-based dressing. Interestingly, a more than 8-log reduction in total bacteria count was observed with the ASP-1 PU dressing as compared to the gauze controls. Of note, the delivery of the peptide from the dressing proved to be much more efficient than a peptide solution containing the same total amount of ASP-1, and moderately more efficient than the silver-based dressing. Unfortunately, in that study only total bacterial count was determined, impeding the evaluation of whether the antibacterial action of the ASP-1 PU dressing was homogenously directed towards the different species within the mixed population. Nevertheless, the results obtained suggest that the used dressing contributed to the stability and localized delivery of the peptide, resulting in higher efficacy in a polymicrobial infection model.

**Figure 3:**
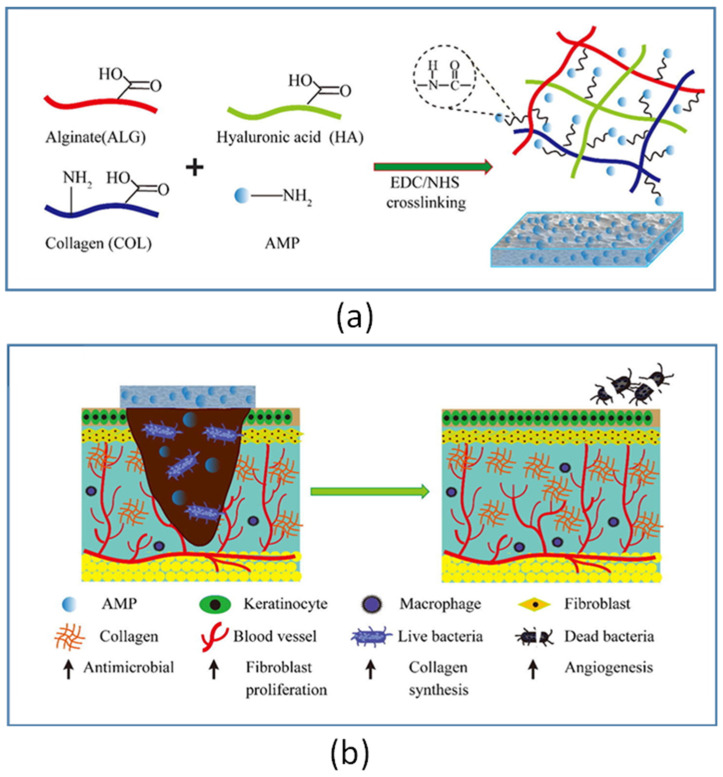
Main steps of the preparation of the ALG/HA/COL-Tet213 wound dressings. (**a**) The AMP Tet213-conjugated ALG/HA/COL wound dressings were prepared using the chemical reaction between the carboxyl groups and the amino groups on ALG, COL, HA, and Tet213; EDC: 1-ethyl-3-(3-dimethylaminopropyl)-carbodiimide; NHS: N-hydroxysuccinimide. (**b**) Multiple biological activities of the Tet213-conjugated ALG/HA/COL. Reproduced from Ref. [53] with permission, with slight modifications.

A different dressing, consisting of alginate (ALG), hyaluronic acid (HA), and collagen (COL), was recently used for chemically cross-linking the AMP Tet213 (Figure 3) [53]. In vitro drug release studies revealed that there was a burst release of Tet213 from the ALG/HA/COL-AMP dressings within the first day of incubation, followed by a sustained release of the peptide for 14 days. When tested in a rat model of *Escherichia coli–S. aureus* mixed wound infection, the ALG/HA/COL-Tet213 dressing accelerated the skin wound closure and healing as compared to the ALG/HA/COL and gauze controls. Furthermore, while at day 4 the number of bacteria per wound in the gauze group was approximately 4.2 × 10^4^ for *E. coli* and 1.8 × 10^7^ for *S. aureus*, in the ALG/HA/COL-Tet213 group this number was significantly reduced, reaching ~0 CFU/wound for *E. coli* and 45 CFU/wound for *S. aureus*, indicating a broad-spectrum activity of the dressing. Interestingly, at day 7 post-infection, an increased collagen deposition and neo-vascularization was observed in the wounds treated with the ALG/HA/COL-Tet213 dressing as compared to the gauze controls, highlighting the multi-functionality of the AMP dressing. The multi-functionality (i.e., the ability to evoke different kinds of favorable effects) is a clear advantage of many AMPs versus the majority of conventional antibiotics, and makes it possible for an AMP to show efficacy in vivo, despite its modest or absent direct antimicrobial activity against the invading pathogens in standard in vitro microbiological tests. The proline-rich antibacterial peptide A3-APO is a striking example of this. The peptide was tested in a mixed *K. pneumoniae*–*A. baumannii*–*Proteus mirabilis* mice wound infection model [54]. Untreated animals died following 22 h infection with no apparent sign of bacteremia, but became paralyzed, suggesting the involvement of the endotoxin released by the Gram-negative pathogens. In contrast, the A3-APO-treated animals displayed a decreased inflammation of the wound sites and a prolonged survival, despite the fact that the peptide was virtually inactive in vitro against the three strains used. A3-APO was found to stimulate the secretion of the anti-inflammatory cytokines IL-10 and IL-4 by peripheral blood mononuclear cells, suggesting that its protective role might be due, at least partially, to the prevention of inflammation at the site of infection [54].

### 2.2. Respiratory Infections

Lung infections are often polymicrobial, as seen in patients suffering from ventilator-associated pneumonia, CF, non-CF bronchiectasis or chronic obstructive pulmonary disease [2,3].

#### 2.2.1. Conventional Therapy

Conventional approaches to treating polymicrobial lung infections consist of the administration of broad-spectrum antimicrobials largely aimed at targeting “traditional” pathogens, such as *P. aeruginosa*, *S. aureus*, *H. influenzae*, and *B. cepacia complex* [4]. In addition to these species, other bacterial, fungal or even viral pathogens, typically isolated concurrently in CF sputum specimens, may greatly influence the progress of the infection and the response to antimicrobial therapy (see Section 3 and Section 4). The consideration of the complexity of the lung community may help to explain why conventional therapies are often less effective than one could expect based on in vitro susceptibility testing. In addition, considering that many microbial species are difficult to culture [82], broad-spectrum antimicrobial agents (e.g., AMPs with antibacterial, antiviral and antifungal activity) could represent a valuable option.

#### 2.2.2. AMP-Based Therapy

Host AMPs represent key elements in the innate defense of the lung, with defensins and cathelicidins being the peptide families most represented in the airway secretions [83]. They can contribute to host defense in the lung by killing the pathogens as well as by modulating the host inflammatory response. These favorable properties have stimulated investigations on their exogenous administration to prevent/treat infections [84,85,86]. Only a few AMPs have been tested in lung polymicrobial infections. One of these peptides is the Tachyplesin III, a β-sheet peptide from the hemocytes of the horseshoe crab (*Tachypleus tridentatus*), which has been tested in bacterial co-infection pneumonia [55]. As compared to mono-bacterial infection, the intranasal co-infection of mice with MDR *P. aeruginosa* and *A. baumannii* caused a more serious disease, with increased pro-inflammatory cytokines (IL-1β, IL-6, TNF-α) and chemokines (MCP-1/MIP-2) and reduced survival. The pretreatment of mice with a single dose of Tachyplesin III (10 mg/kg, i.v.) could prolong mice survival and significantly reduce the total bacterial count in the bronchoalveolar lavage fluid, as compared to the untreated or meropenem-treated control mice groups. Interestingly, the peptide was also found to reduce the serum level of the pro-inflammatory cytokines IL-1β, IL-6, and TNF-α, and to decrease inflammatory cell infiltration, vascular leakage, and alveolar disruption in the Tachyplesin III-pretreated group as compared to the co-infected group or the meropenem-treated group. Finally, when tested in vitro, the peptide displayed the ability to enhance the phagocytic function of mouse alveolar macrophages, suggesting that its prophylactic efficacy might be due to multimodal mechanisms of action.

### 2.3. Oral Infections

Another striking example of mixed biofilm-associated bacterial infection is periodontal disease. This comprises a wide range of clinical manifestations that span from a mild and reversible gingivitis to severe, chronic periodontitis, which may lead to the progressive destruction of bone and connective tissue in the periodontal area, with consequent tooth loss [12]. It is largely accepted that specific groups of oral bacteria, such as those belonging to the “red complex” (e.g., *P. gingivalis*, *T. forsythia*, *T. denticola*), play a causative role in the development of periodontitis by invading periodontal tissues and secreting numerous virulence factors [13]. Nevertheless, equally important in the pathogenesis of the disease is the uncontrolled host pro-inflammatory response to bacterial invasion, which includes the upregulation of proinflammatory cytokines, matrix metalloproteinases, and reactive oxygen species, all of which contribute to the tissue damage and loss of teeth commonly associated with periodontitis [87].

#### 2.3.1. Conventional Therapies

Despite the partially specific microbial etiology of periodontitis, the standard treatment of the disease remains highly unspecific, mainly consisting of the mechanical debridement of the root surface [88]. Although successful in many patients, the difficulty of reaching deep and tortuous pockets renders such a therapeutic procedure ineffective in a proportion of diseased sites/patients. Because of these limitations, the systemic or local administration of antibiotics (e.g., amoxicillin, tetracycline, and metronidazole) might be used as an adjunctive therapy to mechanical debridement, although the emerging antibiotic resistance in oral bacteria may limit the treatment’s effectiveness [89].

#### 2.3.2. AMP-Based Therapy

Several AMPs are naturally produced in the oral cavity as part of the innate immune system, and they are believed to greatly contribute to maintaining microbial homeostasis and health status in the oral district [90,91]. As many AMPs have shown good activity against oral bacteria, their use to prevent/treat oral infections seems promising, although their antimicrobial potency in the oral cavity might be challenged by the presence of saliva or crevicular fluid, due to high salt concentration, the presence of proteases of host/bacterial origin, or sequestration by the macromolecules present in such fluids [92,93,94]. Wang and coworkers reported the ability of a synthetic cationic AMP, Nal-P-113, to exert a significant bactericidal activity against oral pathogens, i.e., *Streptococcus gordonii*, *Fusobacterium nucleatum* and *P. gingivalis*, in both planktonic and polymicrobial biofilm states [56]. The peptide is the optimized derivative of another peptide, P-113 (AKRHHGYKRKFH-NH2), in which histidine residues were replaced with the bulky amino acid β-naphthylalanine, resulting in increased salt resistance [95]. Nal-P-113 retained more than 85% integrity after 8 h incubation in phosphate buffered saline (PBS), saliva from healthy donors, brain heart infusion medium, and bovine calf serum. Importantly, at a concentration that only causes slight damage to normal oral cells (1.28 mg/mL), Nal-P-113 was able to eradicate triple strain biofilms of *S. gordoni*, *F. nucleatum* and *P. gingivalis*, while the minimum biofilm eradication concentrations of penicillin and metronidazole were 2 mg/mL and 80 mg/mL, respectively. It is noteworthy that many AMPs (e.g., beta-defensins, human neutrophil defensins, the human cathelicidin LL-37) have shown lipopolysaccharide (LPS)-neutralizing activities against periodontopathogens, causing the inhibition of the IL-1β, IL-8, and intercellular adhesion molecule 1 (ICAM-1) expression induced by LPS from *P. intermedia* and *T. forsythia* in THP-1 cells and human gingival fibroblasts [96]. Altogether, these results suggest that AMPs may be considered as preventive and therapeutic agents against mixed bacterial infections, such as periodontitis, by killing the pathogens as well as by reducing the activity of LPS and disease-associated inflammation.

### 2.4. Sepsis

Sepsis is a serious life-threatening condition characterized by an excessive systemic inflammation following a blood stream infection (BSI). It is a major public health problem and one of the most common cause of death worldwide during hospital stay [97]. Almost 60% of all types of hospital-acquired BSI originate from vascular access devices (catheter-related blood stream infections, CRBSI) [98]. Such devices are widely used, especially in critically ill patients for the administration of fluids, chemotherapy, antibiotics, or nutritional solutions. Although an integral part of modern medical practice, intravascular catheters are prone to colonization by skin microorganisms that eventually develop a biofilm on the foreign body surfaces (external and internal) [99]. As the biofilm matures, single microorganisms or biofilm particles may detach and gain access to the blood stream, leading to a CRBSI. Polymicrobial BSI is gaining epidemiological significance, as it accounts for 5–38% of all BSI and is reported to evolve into deadly sepsis at a higher rate than monomicrobial bacteremia [17,18,100].

#### 2.4.1. Conventional Therapies

Among others, inadequate antimicrobial therapy that fails to target all microorganisms involved (often multi-antibiotic resistant) is a factor associated with increased mortality in polymicrobial sepsis [18]. Furthermore, antibiotic treatment in sepsis may cause the lysis of bacterial cells with the consequent release of cell wall-associated proinflammatory components (e.g., LPS) that, in turn, amplify the inflammatory cascade. Thus, an efficacious therapeutic intervention in polymicrobial sepsis must be broad-spectrum, able to target resistant bacteria, and possibly capable of reducing the sepsis-associated pro-inflammatory response.

#### 2.4.2. AMP-Based Therapy

Su et al. evaluated the potential of Epinecidin-1, an AMP from orange-spotted grouper (*Epinephelus coioides*), in treating polymicrobial sepsis and endotoxemia [57]. Polymicrobial sepsis was induced in mice via cecal ligation and puncture (CLP) [101]. At a period of 30 min from surgery, mice were treated with Epinecidin-1 (50 mg/kg) by intraperitoneal injection, and pathology, immune response and survival rate were evaluated as compared to the control groups (CLP + saline injected mice). Mice treated with Epinecidin-1 displayed an increased survival rate as compared to the CLP + saline group. In addition, Epinecidin-1 injection markedly improved CLP-induced lung injury and immune cell accumulation, and decreased the level of systemic inflammatory markers (i.e., IL-6, IL-12, IL-18, and TNF-α) and peritoneal bacterial load. Similar protective effects were also observed in mice following LPS-induced endotoxemia. As in the case of periodontitis, the potential of AMPs to target both bacterial proliferation and the inflammatory response may be a benefit over classical antibiotics.

A murine polymicrobial sepsis model was also employed to assess the therapeutic potential of three newly developed synthetic AMPs, specifically designed to bind the lipid A part of endotoxins [58]. Among them, peptide 19–2.5 (Pep2.5) was found to significantly increase the physical activity of mice, evaluated by means of a predefined scoring system ranging from 1 (healthy) to 5 (agony), as compared to control mice, following 24 h of CLP-induced sepsis. Furthermore, continuous Pep2.5 infusion reduced the markedly elevated IL-6, IL-10 and monocyte chemoattractant protein serum levels in septic animals and CD14 mRNA expression in the heart, lung and spleen, suggesting a potential of the peptide in the treatment of sepsis. Interestingly, the same peptide was found to attenuate the cardiac dysfunction, often associated with sepsis, in a murine polymicrobial sepsis model by preventing the downregulation of cardiac sarcoplasmic reticulum Ca2+-ATP-ase (SERCA2), highlighting the multimodal action of many AMPs [59].

### 2.5. Infections of the Lower Female Reproductive Tract

Bacterial vaginosis (BV) is a common mucosal infection that affects a large percentage of women of reproductive age. It is characterized by a shift from a *Lactobacillus*-dominated commensal flora towards a mixed flora of facultative and obligate anaerobic bacteria [102].

#### 2.5.1. Conventional Therapy

The standard treatment is the administration of metronidazole, clindamycin or tinidazole orally or intravaginally. However, the treatment with these antibiotics is associated with high levels of failure and recurrence rates due to antibiotic resistance, inability to eradicate the polymicrobial biofilms, or failure to reestablish acidic pH and the *Lactobacillus*-dominated commensal flora [7]. Therefore, alternative strategies to replace or to be combined with standard therapies to prevent and treat BV more efficiently are under evaluation.

#### 2.5.2. AMP-Based Therapy

Zhu and coworkers tested the therapeutic potential of the AMP HPRP-A2 in combination with chlorhexidine acetate (CHA) in a rat vaginitis infection model [60]. They infected the animals intravaginally with a 1:1 suspension of *E. coli* and *S. aureus*. After 8 days of treatment with HPRP-A2, CHA or their combination, the vaginal bacterial count was evaluated. In both low-dose and high-dose treatment groups a statistically significant reduction in the CFU counts of both bacterial species was observed as compared to the control animals. The highest rate of inhibition was observed in the animals treated with the HPRP-A2-CHA combination. For instance, as compared with the untreated controls, the reduction in the CFU count of *E. coli* and *S. aureus* treated with a high dose of HPRP-A2 or CHA alone ranged from 56.9 to 67.3%, while their combination reached an inhibition of 99.9%, stressing the possibility of successfully combining AMPs with conventional drugs to obtain a synergistic therapeutic effect.

## 3. Bacteria–Fungi Mixed Infections

Bacteria and fungi often co-exist in the same environmental and body niches, forming polymicrobial biofilms and establishing interkingdom interactions whose importance in the pathogenesis of many infections is progressively emerging [1,103]. Bacteria–fungi mixed infections may occur in several body districts, including the skin, the oral cavity, the lung, the gastrointestinal tract, the lower female reproductive tract, as well as the blood stream often as a consequence of the mixed colonization of intravenous access devices [19,103,104]. Bacteria and fungi can interact through several means in mixed biofilms. These include coaggregation, the mutual induction of resistance to antibacterial/antifungal drugs, the reciprocal modulation of invasive properties or the expression of virulence factors [103]. One of the most studied bacteria–fungi interactions is that which establishes between *Candida* and *Staphylococcus*. It has been reported that *S. aureus* binds in high numbers to *C. albicans* hyphae, and that the bacterium shows an increased resistance to vancomycin in the presence of the yeast [27]. *C. albicans* can also interact with *P. aeruginosa*, and such an interaction favors the development of candidiasis in a mouse burn model [105]. This effect was ascribed to the production of the virulence factor elastase by *P. aeruginosa*, which may have promoted tissue damage and facilitated *C. albicans* dissemination from the skin. Bacteria–fungi interactions are also very relevant in the oral cavity, where *C. albicans* interact at different levels with streptococci (e.g., *S. mutans*, *S. gordonii*) or periodontal pathogens (e.g., *P. gingivalis*, *Aggregatibacter actinomycetemcomitans*) with a synergistic effect on colonization and the pathogenesis of oral diseases [103].

### 3.1. Conventional Therapy

The chemotherapeutic treatment of bacteria–fungi polymicrobial biofilms requires a combination of antibiotics and anti-fungal agents, but it has been reported that such a combination strategy shows poor efficacy, usually failing in c.a. 70% of infections [27]. In addition, as the contribution of fungi in a clinical infection is often missed during microbiological diagnosis by standard culture methods, the patient is treated for mono-species bacterial infection and the cure is not obtained [103]. Thus, antimicrobial agents with broad inter-kingdom activity would help in targeting this type of infection.

### 3.2. AMP-Based Therapy

As several AMPs exert both antibacterial and antifungal activity [106,107,108], they could serve in this area. For instance, de Alteriis and coworkers recently tested the activity of the membranotropic peptide gH625 and its derivative gH625-GCGKKK in impairing polymicrobial biofilms formed by *C. tropicalis* and the Gram-positive *S. aureus* or the Gram-negative *Serratia marcescens* [61]. By using scanning electron microscopy (SEM) and differential fluorescence staining, they demonstrated in mature mixed biofilms the presence of a dense network of fungal (both yeast-like and elongated forms) and bacterial cells, surrounded by an abundant extracellular polymeric substance. When tested in a biofilm prevention assay, gH625-GCGKKK showed a higher inhibitory capacity than gH625, inhibiting the biofilm formation of both fungal–bacterial combinations by 80% at a concentration lower that its MIC (50 µM). Unfortunately, inhibition was only evaluated as the reduction in biofilm biomass via crystal violet staining, and not by differential CFU count on selective media, impeding evaluation in the mixed biofilm if the peptide-inhibitory activity was preferentially directed against the fungus or the bacteria. The two peptides were also active in eradicating biofilms of *C. tropicalis* and *S. marcescens*, or *C. tropicalis* and *S. aureus*, pre-formed on both polystyrene 96-well plates and medical-grade silicone plates. As gH625 is also able to act as a cell-penetrating peptide [109], the authors suggest a therapeutical potential of these peptides in destroying pre-existing biofilms, and as carriers for other anti-infective agents for a synergistic anti-biofilm effect.

Gupta and coworkers [62] performed another interesting and exhaustive study. They screened 20 cholic acid-peptide conjugates (CAPs) for their antimicrobial activity against different Gram-positive bacterial and fungal strains. Among them, the valine–glycine-derived CAP-3 exhibited a broad antimicrobial spectrum, inhibiting the growth of both *S. aureus* and *C. albicans* at the concentrations of 8 and 4 µM, respectively. Importantly, the same CAP showed a higher selectivity towards microbial cells than mammalian epithelial cells, did not induce resistance in either *S. aureus* or *C. albicans* following multiple passages in vitro, and was active towards persisters and stationary phase cells, which are usually highly tolerant to conventional antibiotics. CAP-3 showed a striking ability to reduce interkingdom polymicrobial biofilms formed by *S. aureus* and *C. albicans*, causing a 4- to 5-log reduction in the CFU of both species at 32 µM. Of note, the CAP-3 treatment alone was as effective as the combination of ciprofloxacin and fluconazole used as the positive control. At the same concentration of 32 µM, CAP-3 significantly reduced the polymicrobial biofilms pre-formed on silicon catheters, while CAP-3-coated catheters prevented the formation of the same biofilms. Finally, the therapeutic efficacy of CAP-3 was confirmed in mice, in two different infection models. In the first one, the peptide was administered three times per day for three days to skin-injured neutropenic mice, wound-infected with both *S. aureus* and *C. albicans*. The quantification of the microbial burden on day four by differential CFU count revealed a 2-log decrease in both *S. aureus* and *C. albicans* colonies in mice treated with CAP-3, as compared to the untreated control mice. In the second model, a combination of bioluminescent *S. aureus* and *C. albicans* was used to infect CAP-3-coated catheters subcutaneously inserted in BALB/C mice. The quantification of the bioluminescence using in vivo imaging showed a significant reduction in both bacterial and fungal load in mice with CAP-3-coated catheters as compared to control mice, highlighting the efficacy of CAP-3 in preventing catheter-related dual species infections.

## 4. Bacteria–Virus Mixed Infections

Interkingdom interactions are not limited to those between bacteria and fungi. Emerging evidence suggests that complex interplay may also be established between bacteria and viruses, significantly impacting the outcome of the mixed infection and the response to antimicrobial/antiviral therapy [110]. Although bacteria are not permissive to eukaryotic virus infection, they can promote viral fitness by enhancing virion stability, promoting the infection of eukaryotic cells, or increasing coinfection rates. On the other hand, viruses binding to bacteria may promote bacterial adherence to eukaryotic cells [110].

### 4.1. Conventional Therapy

Our knowledge of bacteria–virus interactions in human infections is still limited. Clinical studies suggest that such interactions may occur in patients with CF where *P. aeruginosa* and respiratory syncytial virus (RSV) are the two main pathogens [111]. RSV infection has been reported to promote the biofilm mode of growth in *P. aeruginosa* [112], and to increase bacterial resistance to frontline traditional antibiotics [63].

### 4.2. AMP-Based Therapy

The cationic AMP WLBU2, a 24-residue peptide composed of only arginine, valine and tryptophan, was recently tested against mature biofilms of *P. aeruginosa* formed on polarized bronchial epithelial cells pre-infected with RSV [63]. After as little as 1 h of treatment, the peptide at 50 µM reduced the biofilm-associated *P. aeruginosa* burden by approximately 10-fold, while in the same conditions the human cathelicidin LL37 was completely inactive. Fluorescence microscopy experiments demonstrated that the reduction in biofilm biomass was around 70% after 1 h of treatment with 10 µM WLBU2. Furthermore, the peptide treatment did not alter either the endogenous expression of AMPs or the cytokine and chemokine gene expression by bronchial epithelial cells, suggesting that the antibiofilm effect was due to a direct interaction of the peptide with bacteria, and not to its ability to stimulate mechanisms of clearance by airway epithelium. Importantly, WLBU2 was also able to inhibit viral infectivity, demonstrating the potential of engineered AMPs to act as cross-kingdom single-molecule combination therapies. Rollins-Smith and coworkers recently proposed another example of such a type of application [64]. They reported that a number of caerin 1 AMPs, derived from Australian amphibians, inhibit in vitro the infectivity of HIV and its transfer from dendritic cells to T cells. Two of these AMPs (caerin 1.9 and 1.10) were also found to inhibit the growth of *Neisseria lactamica*, a surrogate for the pathogenic *N. gonorrhoeae*, disclosing their potential in the simultaneous prevention/treatment of two major sexually transmitted infections.

## 5. Single- or Multiple-Targeted AMPs to Discriminate Pathogens within Mixed Communities of Beneficial Bacteria

The importance of polymicrobial infections is increasingly being recognized not only for the multiple interactions that are established among potential pathogens, but also for the interplay among pathogens and members of the commensal flora. The latter greatly contributes to the maintenance of host health status by establishing competitive interactions with pathogens or by inhibiting their virulence, including biofilm formation. Thus, the possibility of preserving the protective normal flora while targeting pathogenic bacteria in the mixed community would be a highly desirable property of innovative antimicrobial agents when the etiology of the infection is known. Unlike most antibiotics that may lose activity if their basic structure is modified, AMPs are particularly prone to molecular alteration. They can be optimized by altering their primary sequence through the incorporation or deletion of hydrophobic or charged amino acids, which has been shown to affect their selectivity for Gram-positive, Gram-negative or fungal membranes [113,114,115,116]. The killing of specific pathogens within a mixed bacterial population has been achieved in vitro by AMPs, referred to as specifically targeted antimicrobial peptides (STAMPs). These are polyfunctional molecules where a domain endowed with antimicrobial activity is combined through a flexible linker with other domains specifically designed to target one or more pathogenic species. For instance, the STAMP C16G2 (TFFRLFNRSFTQALGKGGGKNLRIIRKGIHIIKKY) consists of a region (C16, amino acids 1 through 16) specifically targeting *S. mutans*, a bacterial species playing a primary role in the onset of dental caries, attached by means of three glycine residues to a novispirin-derived AMP that represents the “killing” region (G2, amino acids 20 through 35) [117]. C16G2 has been demonstrated to specifically eliminate *S. mutans*, and not other oral streptococci, in both planktonic- and saliva-derived biofilms. Furthermore, multispecies biofilms from which *S. mutans* has been eliminated through C16G2 treatment have been shown to resist colonization from exogenous *S. mutans*. The rapid mechanism of action of C16G2, which kills *S. mutans* within one minute of exposure, and its solubility in aqueous solution have suggested its employment as a mouth rinse. A pilot clinical study on 12 subjects demonstrated that a single rinse with C16G2 was able to cause a statistically significant reduction in *S. mutans* burden in plaque and saliva, as compared to a placebo, with minimal impact on total plaque bacteria. Such a reduction was associated with higher plaque pH, lower acid production and the prevention of enamel demineralization [118]. Other STAMPs have been designed to specifically target *Pseudomonas* spp. [119], *P. aeruginosa* [48] or *P. aeruginosa* + *S. mutans* (“dual-targeted” antimicrobial peptide) [120], demonstrating high specificity when tested against mixed cultures. Interestingly, the aforementioned peptide caerin 1.9 with anti-HIV and anti-*N. lactamica* activity was also found to be inactive against two lactobacilli species (*Lactobacillus rhamnosus* and *L. crispatus*), which are normal inhabitants of vaginal flora with a recognized protective effect in the vaginal environment [64]. Thus, AMPs appear not only as highly versatile molecules with the potential to be used as a broad-spectrum mono-therapy in infections with probable mixed etiologies, but also as highly specific narrow-spectrum tools able to target individual pathogens while leaving beneficial bystander flora unaffected.

## 6. AMP Mimetics against Polymicrobial Infections

A few AMP mimetics have been evaluated as antimicrobials against polymicrobial infections (Table 3) [65,66,67]. Of note, AMP mimetics may help to overcome some of the drawbacks that still limit the therapeutic use of many AMPs. This is the case of peptoids, oligo N-substituted glycines in which the side chains are appended to the nitrogen atom of the peptide backbone, rather than to the α-carbons (as they are in amino acids) [121]. Peptoids exert many features of AMPs, but show an increased resistance to proteolytic degradation [122]. Among a library of 18 linear peptoids, three (peptoids 5, 7 and 17) were recently tested against mixed-species biofilms of *C. albicans* and either *S. aureus* or *E. coli* [67]. Peptoid 17 (NahNspeNspe)_3_ was identified as the most promising candidate. It exhibited low toxicity towards HepG2 epithelial and HaCaT keratinocyte cell lines. Furthermore, at 10 µM, it displayed significant activity against *S. aureus* in a mixed-species biofilm with *C. albicans*, and showed good activity against both species in the *C. albicans* and *E. coli* biofilm at higher concentrations. It is noteworthy that *C. albicans* appeared to be less susceptible to peptoid 17 when in a biofilm with *S. aureus* than in a mono-species biofilm, but this did not appear to be the case with *E. coli*. Such an observation suggests that it might be worth planning future studies to evaluate the impact of polymicrobial cultures on the susceptibility of individual species to AMPs or peptoids.

## 7. Potential Difficulties Arising in the Use of AMPs against Mixed Infections

Despite the numerous advantages underlined above, difficulties may arise when using AMPs against mixed infections. Such difficulties add to the known obstacles that still limit the therapeutic use of AMPs, which include susceptibility to protease degradation, sequestration by biological fluids, inactivation by physiological concentrations of salts, and potential toxicity towards eucaryotic cells, adding complexity to the development of AMP-based therapeutics. For example, when evaluating the use of AMPs against mixed infections, the possible emergence of community-based mechanisms of resistance to AMP candidates or the need to increase doses to control the infection should be considered (Table 2).

In a polymicrobial biofilm, the EPS produced by an individual species may rescue other members of the community from attack by AMPs. A major role of EPS in biofilm resistance to AMPs has been extensively highlighted in a previous review by us [37]. Cationic AMPs may be sequestered by negatively charged EPS (e.g., *P. aeruginosa* alginate), or rather repulsed by positively charged EPS (e.g., the *S. aureus* polysaccharide intercellular adhesin-PIA- at neutral pH), with a consequent decrease in antibiofilm activity [37]. The contribution of this mechanism to AMPs resistance in polymicrobial communities is still largely unknown. Interestingly, the expression of alginate was found to increase when *P. aeruginosa* and *Stenotrophomonas maltophilia* were grown in a mixed biofilm, suggesting that this mechanism might be important in polymicrobial environments [123]. Another study reported that a mixed population of mucoid (alginate over-producers) and non-mucoid *P. aeruginosa* variants exhibits enhanced resistance to the host AMP LL-37 [124]. The exogenous addition of alginate to non-mucoid variants rescued the bacteria from LL-37 killing, suggesting that alginate production by mucoid isolates may represent a shared benefit in mixed communities of mucoid and non-mucoid *P. aeruginosa*, often co-existing in CF lungs. Psl, a further major polysaccharide of the *P. aeruginosa* biofilm matrix, was reported to provide a generic first line of defense toward different antibiotics/AMPs during the initial stages of biofilm development [125]. When antibiotic-sensitive “non-producing” cells lacking Psl were mixed with Psl-producing strains, the former could gain tolerance to antibiotic treatment. Psl-mediated protection was extendible also to *E. coli* and *S. aureus*, which became tolerant to the cyclic AMP colistin and to tobramycin, respectively, in mixed biofilms [125].

Another potential mechanism of AMP-resistance occurring in polymicrobial biofilms could be the induction of a phenotypic switch in specific members of the community by exoproducts released into the environment by cohabitant microbial species. As reported in the introduction, it has been well documented that *P. aeruginosa* exoproducts may induce, in *S. aureus*, the formation of SCVs [30]. Besides being intrinsically resistant to conventional antibiotics, such phenotypic variants have been reported to show decreased susceptibility towards a number of naturally occurring AMPs, including the human beta-defensin (hBD)-2 and -3, RNase 7, and LL-37 [126]. Thus, the phenotypic switch induced by a community member could represent an obstacle to the therapeutic use of synthetic AMPs against polymicrobial infections, as well as a mechanism of evasion by naturally occurring AMPs as part of the innate immune system.

Ryan et al. [26] have described a different mechanism of exoproduct-mediated tolerance to AMPs in mixed infections. They demonstrated that *S. maltophilia* produce a diffusible signal factor (DSF) that influences biofilm formation and polymyxin tolerance in *P. aeruginosa*. Such a response in *P. aeruginosa* is mediated by a sensor kinase (PA1396) that leads to increased levels of several proteins with roles in bacterial stress tolerance, including those implicated in resistance to cationic AMPs. *P. aeruginosa* and *S. maltophilia* co-inhabit a number of environmental niches, including the lungs of CF patients. Thus, in the presence of DSF-producing *S. maltophilia*, *P. aeruginosa* could gain tolerance to host AMPs or to AMPs used as therapeutic agents, including polymyxin E, which represents a last-resort drug for MDR *P. aeruginosa* treatment.

AMP-resistance could also be conferred by one species to other species sharing the same microenvironment through protease production. Important pathogens, such as *P. aeruginosa* or *P. gingivalis*, produce high levels of proteases as virulence factors [127,128]. In a polymicrobial biofilm infection, protease high-producing members could protect themselves, and also low- or no-producing neighbors, from AMP killing (e.g., *P. aeruginosa* in polymicrobial lung infections or *P. gingivalis* in mixed oral infections, such as periodontitis).

Finally, the vicinity of bacterial cells within a mixed community may favor the inter-species exchange of genetic elements, some of which might be involved in AMP-resistance (e.g., genes encoding for efflux pumps or for proteases) [129].

The dose of AMPs required to target mixed infections is another important issue. In principle, if individual members of a community show different degrees of susceptibility to an AMP candidate, a concentration at least able to affect the least susceptible member needs to be selected. Such a concentration is likely to increase further as the number of community members increases. For instance, the Nal-P-113 peptide (Table 3) was reported to have minimum biofilm eradication concentration (MBEC) values of 0.32 mg/mL against single strain biofilms of *F. nucleatum* and *P. gingivalis*, 0.64 mg/mL against dual-strain biofilms (*F. nucleatum* + *P. gingivalis*), and 1.28 mg/mL against triple-strain biofilms (*F. nucleatum* + *P. gingivalis* + *S. gordonii*) [56]. In addition to strain number, different structural features between mono-microbial and polymicrobial biofilms are also likely to play a role. As the bactericidal mechanism of many AMPs relies on their ability to permeabilize and/or form pores within cytoplasmic membranes (mostly prokaryotic but also eukaryotic), safety concerns may arise at high doses.

## 8. Conclusions and Future Research Challenges

The rising appearance and rapid spread of microbial pathogens highly or even pan-resistant to available drugs calls for the discovery of suitable alternatives to current antibiotics. This is particularly true for polymicrobial infections whose treatment poses additional hurdles as compared to mono-microbial infections. AMPs have received great attention as new antimicrobials due to their broad activity spectrum, rapid killing kinetics, unique mechanisms of action, and low tendency to induce resistant variants [130,131]. Nevertheless, they have shown limitations in pharmaceutical development due to potential toxicity, low stability and high manufacturing costs. Because of these limitations, only a few AMPs have been tested in Phase III clinical trials, and even less have been approved for clinical use [132]. Nonetheless, several strategies seem promising in overcoming the limitations of AMPs, including the introduction of non-natural amino acids, cyclization, the optimization of physicochemical characteristics, biosynthesis in suitable recombinant expression systems, and the use of liposomal formulations or adequate delivery systems [131].

Synthetic AMPs may represent a promising option for the treatment of polymicrobial infections, but their therapeutic potential in such types of infections has been relatively poorly investigated. Future work in this field should be directed towards investigating whether individual microbial species alter the sensitivity of other microbial species to AMPs when grown in mixed cultures, similarly to what has been described for many antibiotics. It would also be important to test whether AMP activity in mixed populations is preferentially directed towards individual species and/or vary at different ratios of the co-infecting species. We expect that when directed towards a mixed population of bacteria–bacteria or bacteria–fungal species, AMPs may preferentially interact with specific members due to the higher degree of affinity towards their surface as compared to neighboring members, with the possibility of altering the population composition. Different killing kinetics towards single polymicrobial members might also be possible, with consequences for the population dynamics and, possibly, for the pathogenesis of an infection, which needs to be carefully evaluated. It is also likely that affinity towards microbial surfaces and killing kinetics are influenced by local conditions characterizing different body sites (e.g., temperature, pH, ionic strength, presence of blood, plasma, serum, lung fluids, urine, wound exudate, etc.). Thus, systematic studies addressing the dynamics of individual species within a mixed population challenged with AMPs in different experimental in vivo conditions/in vivo models are desired to better assess the AMP therapeutic potential in mixed infections.

In conclusion, the data available to date for AMPs as a potential treatment for hard-to-treat polymicrobial infections are encouraging, but much fine-tuning needs to be done, before we will be able to successfully exploit the potential of AMPs for these kinds of therapeutic applications.

## Figures and Tables

**Table 1:** Examples of infections with possible polymicrobial etiology and species involved.

Types of Infections with Possible Polymicrobial Etiology	Common Species Involved	References
Lung infections in cystic fibrosis	*Pseudomonas aeruginosa*, *Staphylococcus aureus*, *Haemophilus influenzae*, *Burkholderia cepacia* complex, *Candida albicans*, respiratory syncytial virus	[2,3,4]
Chronic wounds (wound burn infections, diabetic wound infections)	*S. aureus*, coagulase-negative staphylococci, *P. aeruginosa*, *Escherichia coli*, *Klebsiella* spp., *Enterobacter* spp., *Enterococcus* spp. *beta-hemolytic streptococci*, *Candida* spp.	[5,6]
Vaginosis	*Gardnerella vaginalis*, *Atopobium vaginae*, *Peptostreptococci*, *Prevotella* spp., *Mobiluncus* spp., *Mycoplasma* spp., *Ureaplasma urealyticum*, *Fusobacterium nucleatum*, *E. faecalis*	[7,8]
Prostatitis	*Chlamydia trachomatis*, *U. urealyticum*, *Mycoplasma hominis*, *Trichomonas vaginalis*, *E. coli*, Enterococci	[9]
Otitis media	*Streptococcus pneumoniae*, *H. influenzae*, *Moraxella catarrhalis*	[10]
Urinary tract infections	*E. coli*, *Proteus mirabilis*, *E. faecalis*, *K. pneumoniae*, *P. aeruginosa*	[11]
Periodontitis	*Porphyromonas gingivalis*, *Tannerella forsythia*, *Treponema denticola*	[12,13]
Dental caries	*S. mutans*, *C. albicans*	[14]
Medical device-related infections	Coagulase-negative Staphylococci, *S. aureus*, *E. faecalis*, *P. aeruginosa*, *C. albicans*, *K. pneumoniae*	[15,16]
Sepsis following dissemination	*Enterobacteriaceae*, non-group A streptococci, anaerobic bacteria, Staphylococci, *Pseudomonas* spp. *Candida* spp.	[17,18,19]

**Table 2:** Possible advantages and limits of AMPs against polymicrobial biofilm-associated infections as compared to conventional antibiotics.

Property	AMPs	Conventional Antibiotics
Activity spectrum	Generally broad (directed against Gram-positive, Gram-negative, fungi and virus), and possibly able to accomplish a one-molecule combination strategy	Generally narrow, especially last resort antibiotics
Anti-persister activity	Demonstrated for many AMPs	None or poor
Immuno-modulatory capacity	Demonstrated for many AMPs	None or poor
Wound-healing activity	Demonstrated for many AMPs	None or poor
Prone to manipulations	Easy to manipulate to improve antimicrobial activity/reduce toxicity	Difficult to manipulate
Activity against beneficial flora	Possibly able to target AMPs against specific pathogens, leaving undisturbed the normal flora	Active against beneficial flora
Induction of resistance	Generally low; in some cases induction of resistance after several passages in vitro. In the case of polymicrobial infections, possibility of insurgence of community-based AMP-resistance mechanisms	Resistance easily induced. In the case of polymicrobial infections, interspecies interactions may affect the antibiotic susceptibility of individual organisms.
Stability in biological fluids	Generally low unless modifications are made	Generally high
Active concentrations	The need to use increased concentrations as compared to mono-species biofilms has been reported with a consequent risk of cytotoxicity	Therapeutic concentrations against susceptible strains highly optimized
Approval by drug agencies	Difficult; only very few AMPs approved for clinical use	Approval of new antibiotics is slower than needed. Only few large pharmaceutical companies have ongoing antibiotic discovery programs

**Table 3:** Examples of AMPs tested against mixed infections.

AMPs	Sequence ^a^ or Molecular Formula	Co-Infecting Species	Type of Application/Infection Model	Ref.
DRGN-1	PSKKTKPVKPKKVA	*P. aeruginosa* and *S. aureus*	In vitro co-infection model and mouse model of wound infection	[49]
Pexiganan-nisin (dual-AMP)	GIGKFLKKAKKFGKAFVKILKK-NH_2_ C_143_H_230_N_42_O_37_S_7_	*S. aureus* and *P. aeruginosa*	Dual AMP biogel/collagen three-dimensional (3D) model	[50]
CST sulfate salt TP-I-L CIT-1.1 TEMP-A	C_53_H_102_N_16_O_17_S KWCFRVCYRGICYRRCR-NH_2_ GLFDVIKKVASVIGGL-NH_2_ FLPLIGRVLSGIL-NH_2_	*S. aureus* and *P. aeruginosa*	In vitro co-infection model	[51]
ASP-1	RRWVRRVRRWVRRVVRVVRRWVRR	*S. aureus*, *A. baumannii*, *K. pneumoniae*, and *P. aeruginosa*	hydrophilic polyurethane (PU)-based dressing/in vitro co-infection model	[52]
Tet213	KRWWKWWRRC	*E. coli* and *S. aureus*	Peptide-immobilized ALG/HA/COL wound dressings and rat model of wound infection	[53]
A3-APO	[(1-amino-cyclohexane carboxylic acid-RPDKPRPYLPRPRPPRPVR)_2_-2,4-diamino-butyric acid]-NH_2_	*K. pneumoniae*, *A. baumannii*, and *P. mirabilis*	mouse model of wound infection	[54]
Tachyplesin III	KWCFRVCYRGICYRKCR-NH_2_	*P. aeruginosa* and *A. baumannii*	Mouse model of bacterial co-infection pneumonia	[55]
Nal-P-113	AKR-Nal-Nal-GYKRKF-Nal-NH_2_	*F. nucleatum*, *S. gordonii*, and *P. gingivalis*	In vitro artificial saliva-coated hydroxyapatite co-infection model	[56]
Epinecidin-1	GFIFHIIKGLFHAGKMIHGLV	Gut microflora	Mouse model of polymicrobial sepsis and LPS-induced endotoxemia	[57]
Pep19-2.5	GCKKYRRFRWKFKGKFWFWG-NH_2_	Gut microflora	Mouse model of polymicrobial sepsis	[58,59]
HPRP-A2	Nα-acetyl-FKKLKKLFSKLWNWK-NH_2_	*E. coli* and *S. aureus*	Rat bacterial vaginitis	[60]
gH625 gH625-GCGKKKK	HGLASTLTRWAHYNALIRAF HGLASTLTRWAHYNALIRAF-GCGKKKK	*C. tropicalis* and *S. marcescens* or *C. tropicalis* and *S. aureus*	In vitro co-infection model	[61]
CAP-3	CA-V_3_	*S. aureus* and *C. albicans*	In vitro co-infection model. Murine wound and catheter infection models	[62]
WLBU2	RRWVRRVRRWVRRVVRVVRRWVRR	*P. aeruginosa* and Respiratory syncytial virus	In vitro co-infection model	[63]
Caerin 1.9	GLFGVLGSIAKHVLPHVVPVIAEKL-NH_2_	HIV and *Neisseria lactamica*	In vitro assay	[64]
Hs02	KWAVRIIRKFIKGFIS-NH_2_ (intragenic antimicrobial peptide-IAP)	*P. aeruginosa* and *S. aureus*	In vitro co-infection model	[65]
guanylated polymethacrylates	synthetic structural mimics of AMPs	*C. albicans* and *S. aureus*	In vitro co-infection model	[66]
Peptoid 5, 7 and 17	poly-N-substituted glycines	*C. albicans* and *S. aureus* or *C. albicans* and *E. coli*	In vitro co-infection model	[67]

^a^ Peptide sequences using the one-letter code for the amino acid residues are shown. CST: colistin; ALG: alginate; HA: hyaluronic acid; COL: collagen; Nal: β-naphthylalanine; CAP: cholic acid-peptide-conjugate.

## References

[1] Maisetta G., Batoni G. (2020). Editorial: Interspecies interactions: Effects on virulence and antimicrobial susceptibility of bacterial and fungal pathogens. Front. Microbiol..

[2] Filkins L.M., O’Toole G.A. (2015). Cystic fibrosis lung infections: Polymicrobial, complex, and hard to treat. PLoS Pathog..

[3] Limoli D.H., Hoffman L.R. (2019). Help, hinder, hide and harm: What can we learn from the interactions between *Pseudomonas aeruginosa* and *Staphylococcus aureus* during respiratory infections?. Thorax.

[4] Khanolkar R.A., Clark S.T., Wang P.W., Hwang D.M., Yau Y.C.W., Waters V.J., Guttman D.S. (2020). Ecological succession of polymicrobial communities in the cystic fibrosis airways. mSystems.

[5] Bertesteanu S., Triaridis S., Stankovic M., Lazar V., Chifiriuc M.C., Vlad M., Grigore R. (2014). Polymicrobial wound infections: Pathophysiology and current therapeutic approaches. Int. J. Pharm..

[6] Serra R., Grande R., Butrico L., Rossi A., Settimio U.F., Caroleo B., Amato B., Gallelli L., de Franciscis S. (2015). Chronic wound infections: The role of *Pseudomonas aeruginosa* and *Staphylococcus aureus*. Expert. Rev. Anti Infect. Ther..

[7] Tomás M., Palmeira-de-Oliveira A., Simões S., Martinez-de-Oliveira J., Palmeira-de-Oliveira R. (2020). Bacterial vaginosis: Standard treatments and alternative strategies. Int. J. Pharm..

[8] Javed A., Manzoor S. (2020). Comparative analysis of bacterial vaginosis microbiota among pregnant and non-pregnant females and isolation of phages against *Enterococcus faecalis*, *Enterococcus faecium*, and *Shigella flexneri* strains. Microb. Pathog..

[9] Skerk V., Schönwald S., Krhen I., Markovinović L., Beus A., Kuzmanović N.S., Kruzić V., Vince A. (2002). Aetiology of chronic prostatitis. Int. J. Antimicrob. Agents.

[10] Bair K.L., Campagnari A.A. (2020). *Moraxella catarrhalis* promotes stable polymicrobial biofilms with the major otopathogens. Front. Microbiol..

[11] Azevedo A.S., Almeida C., Melo L.F., Azevedo N.F. (2017). Impact of polymicrobial biofilms in catheter-associated urinary tract infections. Crit. Rev. Microbiol..

[12] Mehrotra N., Singh S. (2020). Periodontitis. StatPearls [Internet].

[13] Dahlen G., Basic A., Bylund J. (2019). Importance of virulence factors for the persistence of oral bacteria in the inflamed gingival crevice and in the pathogenesis of periodontal disease. J. Clin. Med..

[14] Sridhar S., Suprabha B.S., Shenoy R., Suman E., Rao A. (2020). Association of *Streptococcus mutans*, *Candida albicans* and oral health practices with activity status of caries lesions among 5-year-old children with early childhood caries. Oral Health Prev. Dent..

[15] Kaya E., Tollapi L., Pastore A., Aringhieri G., Maisetta G., Barnini S., Paolicchi A., Batoni G., Esin S. (2020). Comparison of methods for the microbiological diagnosis of totally implantable venous access port-related infections. J. Med. Microbiol..

[16] Donlan R.M. (2001). Biofilms and device-associated infections. Emerg. Infect. Dis..

[17] Zhang Y., Hu A., Andini N., Yang S. (2019). A ‘culture’ shift: Application of molecular techniques for diagnosing polymicrobial infections. Biotechnol. Adv..

[18] Weinstein M.P., Reller L.B., Murphy J.R. (1986). Clinical importance of polymicrobial bacteremia. Diagn. Microbiol. Infect. Dis..

[19] Klotz S.A., Chasin B.S., Powell B., Gaur N.K., Lipke P.N. (2007). Polymicrobial bloodstream infections involving *Candida* species: Analysis of patients and review of the literature. Diagn. Microbiol. Infect. Dis..

[20] Nguyen A.T., Oglesby-Sherrouse A.G. (2016). Interactions between *Pseudomonas aeruginosa* and *Staphylococcus aureus* during co-cultivations and polymicrobial infections. Appl. Microbiol. Biotechnol..

[21] Dalton T., Dowd S.E., Wolcott R.D., Sun Y., Watters C., Griswold J.A., Rumbaugh K.P. (2011). An in vivo polymicrobial biofilm wound infection model to study interspecies interactions. PLoS ONE.

[22] DeLeon S., Clinton A., Fowler H., Everett J., Horswill A.R., Rumbaugh K.P. (2014). Synergistic interactions of *Pseudomonas aeruginosa* and *Staphylococcus aureus* in an in vitro wound model. Infect. Immun..

[23] Limoli D.H., Yang J., Khansaheb M.K., Helfman B., Peng L., Stecenko A.A., Goldberg J.B. (2016). *Staphylococcus aureus* and *Pseudomonas aeruginosa* co-infection is associated with cystic fibrosis-related diabetes and poor clinical outcomes. Eur. J. Clin. Microbiol. Infect. Dis..

[24] Hotterbeekx A., Kumar-Singh S., Goossens H., Malhotra-Kumar S. (2017). In vivo and in vitro interactions between *Pseudomonas aeruginosa* and *Staphylococcus* spp.. Front. Cell Infect. Microbiol..

[25] Lopez-Medina E., Fan D., Coughlin L.A., Ho E.X., Lamont I.L., Reimmann C., Hooper L.V., Koh A.Y. (2015). *Candida albicans* inhibits *Pseudomonas aeruginosa* virulence through suppression of pyochelin and pyoverdine biosynthesis. PLoS Pathog..

[26] Ryan R.P., Fouhy Y., Garcia B.F., Watt S.A., Niehaus K., Yang L., Tolker-Nielsen T., Dow J.M. (2008). Interspecies signalling via the *Stenotrophomonas maltophilia* diffusible signal factor influences biofilm formation and polymyxin tolerance in *Pseudomonas aeruginosa*. Mol. Microbiol..

[27] Harriott M.M., Noverr M.C. (2009). *Candida albicans* and *Staphylococcus aureus* form polymicrobial biofilms: Effects on antimicrobial resistance. Antimicrob. Agents Chemother..

[28] Orazi G., O’Toole G.A. (2017). *Pseudomonas aeruginosa* alters *Staphylococcus aureus* sensitivity to vancomycin in a biofilm model of cystic fibrosis infection. mBio.

[29] Hoffman L.R., Deziel E., D’Argenio D.A., Lepine F., Emerson J., McNamara S., Gibson R.L., Ramsey B.W., Miller S.I. (2006). Selection for *Staphylococcus aureus* small-colony variants due to growth in the presence of *Pseudomonas aeruginosa*. Proc. Natl. Acad. Sci. USA.

[30] Garcia L.G., Lemaire S., Kahl B.C., Becker K., Proctor R.A., Denis O., Tulkens P.M., Van Bambeke F. (2013). Antibiotic activity against small-colony variants of *Staphylococcus aureus*: Review of *in vitro*, animal and clinical data. J. Antimicrob. Chemother..

[31] Li H., Li X., Wang Z., Fu Y., Ai Q., Dong Y., Ju J. (2015). Autoinducer-2 regulates *Pseudomonas aeruginosa* PAO1 biofilm formation and virulence production in a dose-dependent manner. BMC Microbiol..

[32] Mashburn L.M., Jett A.M., Akins D.R., Whiteley M. (2005). *Staphylococcus aureus* serves as an iron source for *Pseudomonas aeruginosa* during in vivo coculture. J. Bacteriol..

[33] Orazi G., O’Toole G.A. (2019). “It takes a village”: Mechanisms underlying antimicrobial recalcitrance of polymicrobial biofilms. J. Bacteriol..

[34] Adam B., Baillie G.S., Douglas L.J. (2002). Mixed species biofilms of *Candida albicans* and *Staphylococcus epidermidis*. J. Med. Microbiol..

[35] Nabb D.L., Song S., Kluthe K.E., Daubert T.A., Luedtke B.E., Nuxoll A.S. (2019). Polymicrobial interactions induce multidrug tolerance in *Staphylococcus aureus* through energy depletion. Front. Microbiol..

[36] Lasa I., Solano C. (2018). Polymicrobial infections: Do bacteria behave differently depending on their neighbors?. Virulence.

[37] Batoni G., Maisetta G., Esin S. (2016). Antimicrobial peptides and their interaction with biofilms of medically relevant bacteria. Biochim. Biophys. Acta.

[38] Riool M., de Breij A., Drijfhout J.W., Nibbering P.H., Zaat S.A.J. (2017). Antimicrobial peptides in biomedical device manufacturing. Front Chem..

[39] Di Luca M., Maccari G., Maisetta G., Batoni G. (2015). BaAMPs: The database of biofilm-active antimicrobial peptides. Biofouling.

[40] Gupta S., Sharma A.K., Jaiswal S.K., Sharma V.K. (2016). Prediction of biofilm inhibiting peptides: An in silico approach. Front. Microbiol..

[41] Sharma A., Gupta P., Kumar R., Bhardwaj A. (2016). DPABBs: A novel in silico approach for predicting and designing. Sci. Rep..

[42] Fallah Atanaki F., Behrouzi S., Ariaeenejad S., Boroomand A., Kavousi K. (2020). BIPEP: Sequence-based prediction of biofilm inhibitory peptides using a combination of nmr and physicochemical descriptors. ACS Omega.

[43] Grassi L., Di Luca M., Maisetta G., Rinaldi A.C., Esin S., Trampuz A., Batoni G. (2017). Generation of persister cells of *Pseudomonas aeruginosa* and *Staphylococcus aureus* by chemical treatment and evaluation of their susceptibility to membrane-targeting agents. Front. Microbiol..

[44] de Breij A., Riool M., Cordfunke R.A., Malanovic N., de Boer L., Koning R.I., Ravensbergen E., Franken M., van der Heijde T., Boekema B.K. (2018). The antimicrobial peptide SAAP-148 combats drug-resistant bacteria and biofilms. Sci. Transl. Med..

[45] Mangoni M.L., McDermott A.M., Zasloff M. (2016). Antimicrobial peptides and wound healing: Biological and therapeutic considerations. Exp. Dermatol..

[46] Grassi L., Pompilio A., Kaya E., Rinaldi A.C., Sanjust E., Maisetta G., Crabbé A., Di Bonaventura G., Batoni G., Esin S. (2020). The anti-microbial peptide (Lin-SB056-1)2-K reduces pro-inflammatory cytokine release through interaction with *Pseudomonas aeruginosa* lipopolysaccharide. Antibiotics.

[47] Gao Y., Fang H., Fang L., Liu D., Liu J., Su M., Fang Z., Ren W., Jiao H. (2018). The Modification and design of antimicrobial peptide. Curr. Pharm. Des..

[48] Kim H., Jang J.H., Kim S.C., Cho J.H. (2020). Development of a novel hybrid antimicrobial peptide for targeted killing of *Pseudomonas aeruginosa*. Eur. J. Med. Chem..

[49] Chung E.M.C., Dean S.N., Propst C.N., Bishop B.M., van Hoek M.L. (2017). Komodo dragon-inspired synthetic peptide DRGN-1 promotes wound-healing of a mixed-biofilm infected wound. npj Biofilms Microbiomes.

[50] Gomes D., Santos R., Soares R.S., Reis S., Carvalho S., Rego P., Peleteiro M.C., Tavares L., Oliveira M. (2020). Pexiganan in combination with nisin to control polymicrobial diabetic foot infections. Antibiotics.

[51] Jorge P., Grzywacz D., Kamysz W., LourencËo A., Pereira M.O. (2017). Searching for new strategies against biofilm infections: Colistin-AMP combinations against *Pseudomonas aeruginosa* and *Staphylococcus aureus* single- and double-species biofilms. PLoS ONE.

[52] Bayramov D., Li Z., Patel E., Izadjoo M., Kim H., Neff J. (2018). A novel peptide-based antimicrobial wound treatment is effective against biofilms of multi-drug resistant wound pathogens. Mil. Med..

[53] Lin Z., Wu T., Wang W., Li B., Wang M., Chen L., Xia H., Zhang T. (2019). Biofunctions of antimicrobial peptide-conjugated alginate/hyaluronic acid/collagen wound dressings promote wound healing of a mixed-bacteria-infected wound. Int. J. Biol. Macromol..

[54] Ostorhazi E., Holub M.C., Rozgonyi F., Harmos F., Cassone M., Wade J.D., Otvos L. (2011). Broad-spectrum antimicrobial efficacy of peptide A3-APO in mouse models of multidrug-resistant wound and lung infections cannot be explained by in vitro activity against the pathogens involved. Int. J. Antimicrob. Agents.

[55] Qi J., Gao R., Liu C., Shan B., Gao F., He J., Yuan M., Xie H., Jin S., Ma Y. (2019). Potential role of the antimicrobial peptide Tachyplesin III against multidrug-resistant *P. aeruginosa* and *A. baumannii* coinfection in an animal model. Infect. Drug Resist..

[56] Wang H.Y., Cheng J.W., Yu H.Y., Lin L., Chih Y.H., Pan Y.P. (2015). Efficacy of a novel antimicrobial peptide against periodontal pathogens in both planktonic and polymicrobial biofilm states. Acta Biomater..

[57] Su B.C., Huang H.N., Lin T.W., Hsiao C.D., Chen J.Y. (2017). Epinecidin-1 protects mice from LPS-induced endotoxemia and cecal ligation and puncture-induced polymicrobial sepsis. Biochim. Biophys. Acta Mol. Basis Dis..

[58] Schuerholz T., Doemming S., Hornef M., Martin L., Simon T.P., Heinbockel L., Brandenburg K., Marx G. (2013). The anti-inflammatory effect of the synthetic antimicrobial peptide 19-2.5 in a murine sepsis model: A prospective randomized study. Crit. Care.

[59] Martin L., Horst K., Chiazza F., Oggero S., Collino M., Brandenburg K., Hildebrand F., Marx G., Thiemermann C., Schuerholz T. (2016). The synthetic antimicrobial peptide 19-2.5 attenuates septic cardiomyopathy and prevents down-regulation of SERCA2 in polymicrobial sepsis. Sci. Rep..

[60] Zhu J., Huang Y., Chen M., Hu C., Chen Y. (2019). Functional synergy of antimicrobial peptides and chlorhexidine acetate against Gram-negative/Gram-positive bacteria and a fungus in vitro and in vivo. Infect. Drug Resist..

[61] de Alteriis E., Lombardi L., Falanga A., Napolano M., Galdiero S., Siciliano A., Carotenuto R., Guida M., Galdiero E. (2018). Polymicrobial antibiofilm activity of the membranotropic peptide gH625 and its analogue. Microb. Pathog..

[62] Gupta S., Thakur J., Pal S., Gupta R., Mishra D., Kumar S., Yadav K., Saini A., Yavvari P.S., Vedantham M. (2019). Cholic acid-peptide conjugates as potent antimicrobials against interkingdom polymicrobial biofilms. Antimicrob. Agents Chemother..

[63] Melvin J.A., Lashua L.P., Kiedrowski M.R., Yang G., Deslouches B., Montelaro R.C., Bomberger J.M. (2016). Simultaneous antibiofilm and antiviral activities of an engineered antimicrobial peptide during virus-bacterium coinfection. mSphere.

[64] Rollins-Smith L.A., Smith P.B., Ledeczi A.M., Rowe J.M., Reinert L.K. (2020). Caerin 1 antimicrobial peptides that inhibit HIV and *Neisseria* may spare protective Lactobacilli. Antibiotics.

[65] Bessa L.J., Manickchand J.R., Eaton P., Leite J.R.S.A., Brand G.D., Gameiro P. (2019). Intragenic antimicrobial peptide Hs02 hampers the proliferation of single- and dual-species biofilms of *P. aeruginosa* and *S. aureus*: A promising agent for mitigation of biofilm-associated infections. Int. J. Mol. Sci..

[66] Qu Y., Locock K., Verma-Gaur J., Hay I.D., Meagher L., Traven A. (2016). Searching for new strategies against polymicrobial biofilm infections: Guanylated polymethacrylates kill mixed fungal/bacterial biofilms. J. Antimicrob. Chemother..

[67] Luo Y., Bolt H.L., Eggimann G.A., McAuley D.F., McMullan R., Curran T., Zhou M., Jahoda P.C., Cobb S.L., Lundy F.T. (2017). Peptoid efficacy against polymicrobial biofilms determined by using propidium monoazide-modified quantitative PCR. ChemBioChem.

[68] Järbrink K., Ni G., Sönnergren H., Schmidtchen A., Pang C., Bajpai R., Car J. (2016). Prevalence and incidence of chronic wounds and related complications: A protocol for a systematic review. Syst. Rev..

[69] Metcalf D.G., Bowler P.G. (2013). Biofilm delays wound healing: A review of the evidence. Burns Trauma.

[70] Harding K. (2015). Innovation and wound healing. J. Wound Care.

[71] Wilson J.R., Mills J.G., Prather I.D. (2005). A toxicity index of skin and wound cleansers used on in vitro fibroblasts and keratinocytes. Adv. SkinWound Care.

[72] Zhang X., Shu W., Yu Q., Qu W., Wang Y., Li R. (2020). Functional biomaterials for treatment of chronic wound. Front. Bioeng. Biotechnol..

[73] Kwiecien K., Zegar A., Jung J., Brzoza P., Kwitniewski M., Godlewska U., Grygier B., Kwiecinska P., Morytko A., Cichy J. (2019). Architecture of antimicrobial skin defense. Cytokine Growth Factor Rev..

[74] Zhang L.J., Gallo R.L. (2016). Antimicrobial peptides. Curr. Biol..

[75] Thapa R.K., Diep D.B., Tønnesen H.H. (2020). Topical antimicrobial peptide formulations for wound healing: Current developments and future prospects. Acta Biomater..

[76] Woodburn K.W., Jaynes J.M., Clemens L.E. (2019). Evaluation of the antimicrobial peptide, RP557, for the broad-spectrum treatment of wound pathogens and biofilm. Front. Microbiol..

[77] Duplantier A.J., van Hoek M.L. (2013). The human cathelicidin antimicrobial peptide LL-37 as a potential treatment for polymicrobial infected wounds. Front. Immunol..

[78] Grassi L., Maisetta G., Esin S., Batoni G. (2017). Combination strategies to enhance the efficacy of antimicrobial peptides against bacterial biofilms. Front. Microbiol..

[79] Christ K., Wiedemann I., Bakowsky U., Sahl H.-G., Bendas G. (2007). The role of lipid II in membrane binding of and pore formation by nisin analyzed by two combined biosensor techniques. Biochim. Biophys. Acta Biomembr..

[80] Sandreschi S., Piras A.M., Batoni G., Chiellini F. (2016). Perspectives on polymeric nanostructures for the therapeutic application of antimicrobial peptides. Nanomedicine.

[81] Piras A.M., Maisetta G., Sandreschi S., Gazzarri M., Bartoli C., Grassi L., Esin S., Chiellini F., Batoni G. (2015). Chitosan nanoparticles loaded with the antimicrobial peptide temporin B exert a long-term antibacterial activity in vitro against clinical isolates of *Staphylococcus epidermidis*. Front. Microbiol..

[82] Burns J.L., Rolain J.M. (2014). Culture-based diagnostic microbiology in cystic fibrosis: Can we simplify the complexity?. J. Cyst Fibros..

[83] Hiemstra P.S. (2007). The role of epithelial beta-defensins and cathelicidins in host defense of the lung. Exp. Lung Res..

[84] Hou M., Zhang N., Yang J., Meng X., Yang R., Li J., Sun T. (2013). Antimicrobial peptide LL-37 and IDR-1 ameliorate MRSA pneumonia in vivo. Cell Physiol. Biochem..

[85] Barlow P.G., Svoboda P., Mackellar A., Nash A.A., York I.A., Pohl J., Davidson D.J., Donis R.O. (2011). Antiviral activity and increased host defense against influenza infection elicited by the human cathelicidin LL-37. PLoS ONE.

[86] Beaumont P.E., McHugh B., Gwyer Findlay E., Mackellar A., Mackenzie K.J., Gallo R.L., Govan J.R., Simpson A.J., Davidson D.J. (2014). Cathelicidin host defence peptide augments clearance of pulmonary *Pseudomonas aeruginosa* infection by its influence on neutrophil function in vivo. PLoS ONE.

[87] Sczepanik F.S.C., Grossi M.L., Casati M., Goldberg M., Glogauer M., Fine N., Tenenbaum H.C. (2020). Periodontitis is an inflammatory disease of oxidative stress: We should treat it that way. Periodontol 2000.

[88] Herrera D., Matesanz P., Bascones-Martínez A., Sanz M. (2012). Local and systemic antimicrobial therapy in periodontics. J. Evid. Based Dent. Pract..

[89] Ardila C.M., Bedoya-García J.A. (2020). Antimicrobial resistance of *Aggregatibacter actinomycetemcomitans*, *Porphyromonas gingivalis* and *Tannerella forsythia* in periodontitis patients. J. Glob. Antimicrob. Resist..

[90] Dale B.A., Fredericks L.P. (2005). Antimicrobial peptides in the oral environment: Expression and function in health and disease. Curr. Issues Mol. Biol..

[91] Brancatisano F.L., Maisetta G., Barsotti F., Esin S., Miceli M., Gabriele M., Giuca M.R., Campa M., Batoni G. (2011). Reduced human beta defensin 3 in individuals with periodontal disease. J. Dent. Res..

[92] Maisetta G., Batoni G., Esin S., Luperini F., Pardini M., Bottai D., Florio W., Giuca M.R., Gabriele M., Campa M. (2003). Activity of human beta-defensin 3 alone or combined with other antimicrobial agents against oral bacteria. Antimicrob. Agents Chemother..

[93] Maisetta G., Batoni G., Esin S., Raco G., Bottai D., Favilli F., Florio W., Campa M. (2005). Susceptibility of *Streptococcus mutans* and *Actinobacillus actinomycetemcomitans* to bactericidal activity of human beta-defensin 3 in biological fluids. Antimicrob. Agents Chemother..

[94] Khurshid Z., Zafar M.S., Naseem M., Khan R.S., Najeeb S. (2018). Human oral defensins antimicrobial peptides: A future promising antimicrobial drug. Curr. Pharm. Des..

[95] Yu H.Y., Tu C.H., Yip B.S., Chen H.L., Cheng H.T., Huang K.C., Lo H.J., Cheng J.W. (2011). Easy strategy to increase salt resistance of antimicrobial peptides. Antimicrob. Agents Chemother..

[96] Lee S.H., Jun H.K., Lee H.R., Chung C.P., Choi B.K. (2010). Antibacterial and lipopolysaccharide (LPS)-neutralising activity of human cationic antimicrobial peptides against periodontopathogens. Int. J. Antimicrob. Agents.

[97] Fleischmann C., Scherag A., Adhikari N.K., Hartog C.S., Tsaganos T., Schlattmann P., Angus D.C., Reinhart K. (2016). International forum of acute care trialists. Assessment of global incidence and mortality of hospital-treated sepsis. Current estimates and limitations. Am. J. Respir. Crit. Care Med..

[98] Crnich C.J., Maki D.G. (2001). The role of intravascular devices in sepsis. Curr. Infect. Dis. Rep..

[99] Bustos C., Aguinaga A., Carmona-Torre F., Del Pozo J.L. (2014). Long-term catheterization: Current approaches in the diagnosis and treatment of port-related infections. Infect Drug Resist..

[100] Zheng C., Zhang S., Chen Q., Zhong L., Huang T., Zhang X., Zhang K., Zhou H., Cai J., Du L. (2020). Clinical characteristics and risk factors of polymicrobial *Staphylococcus aureus* bloodstream infections. Antimicrob. Resist. Infect. Control.

[101] Dejager L., Pinheiro I., Dejonckheere E., Libert C. (2011). Cecal ligation and puncture: The gold standard model for polymicrobial sepsis?. Trends Microbiol..

[102] Tidbury F., Langhart A., Weidlinger S., Stute P. (2020). Non-antibiotic treatment of bacterial vaginosis-a systematic review. Arch. Gynecol. Obstet..

[103] Allison D.L., Willems H.M.E., Jayatilake J.A.M.S., Bruno V.M., Peters B.M., Shirtliff M.E. (2016). *Candida*-bacteria interactions: Their impact on human disease. Microbiol. Spectr..

[104] Peleg A.Y., Hogan D.A., Mylonakis E. (2010). Medically important bacterial–fungal interactions. Nat. Rev. Microbiol..

[105] Neely A.N., Law E.J., Holder I.A. (1986). Increased susceptibility to lethal *Candida* infections in burned mice preinfected with *Pseudomonas aeruginosa* or pretreated with proteolytic enzymes. Infect. Immun..

[106] Kim H., Jang J.H., Kim S.C., Cho J.H. (2014). De novo generation of short antimicrobial peptides with enhanced stability and cell specificity. J. Antimicrob. Chemother..

[107] Lombardi L., Maisetta G., Batoni G., Tavanti A. (2015). Insights into the antimicrobial properties of hepcidins: Advantages and drawbacks as potential therapeutic agents. Molecules.

[108] Luo Y., McLean D.T., Linden G.J., McAuley D.F., McMullan R., Lundy F.T. (2017). The naturally occurring host defense peptide, LL-37, and its truncated mimetics KE-18 and KR-12 have selected biocidal and antibiofilm activities against *Candida albicans*, *Staphylococcus aureus*, and *Escherichia coli* in vitro. Front. Microbiol..

[109] Falanga A., Vitiello M.T., Cantisani M., Tarallo R., Guarnieri D., Mignogna E., Netti P., Pedone C., Galdiero M., Galdiero S. (2011). A peptide derived from herpes simplex virus type 1 glycoprotein H: Membrane translocation and applications to the delivery of quantum dots. Nanomedicine.

[110] Neu U., Mainou B.A. (2020). Virus interactions with bacteria: Partners in the infectious dance. PLoS Pathog..

[111] van Ewijk B.E., van der Zalm M.M., Wolfs T.F., van der Ent C.K. (2005). Viral respiratory infections in cystic fibrosis. J. Cyst. Fibros..

[112] Hendricks M.R., Lashua L.P., Fischer D.K., Flitter B.A., Eichinger K.M., Durbin J.E., Sarkar S.N., Coyne C.B., Empey K.M., Bomberger J.M. (2016). Respiratory syncytial virus infection enhances *Pseudomonas aeruginosa* biofilm growth through dysregulation of nutritional immunity. Proc. Natl. Acad. Sci. USA.

[113] Tossi A., Sandri L., Giangaspero A. (2000). Amphipathic, alpha-helical antimicrobial peptides. Biopolymers.

[114] Muhle S.A., Tam J.P. (2001). Design of Gram-negative selective antimicrobial peptides. Biochemistry.

[115] Batoni G., Casu M., Giuliani A., Luca V., Maisetta G., Mangoni M.L., Manzo G., Pintus M., Pirri G., Rinaldi A.C. (2016). Rational modification of a dendrimeric peptide with antimicrobial activity: Consequences on membrane-binding and biological properties. Amino Acids.

[116] Manzo G., Ferguson P.M., Gustilo V.B., Hind C.K., Clifford M., Bui T.T., Drake A.F., Atkinson R.A., Sutton J.M., Batoni G. (2019). Minor sequence modifications in temporin B cause drastic changes in antibacterial potency and selectivity by fundamentally altering membrane activity. Sci. Rep..

[117] Eckert R., He J., Yarbrough D.K., Qi F., Anderson M.H., Shi W. (2006). Targeted killing of *Streptococcus mutans* by a pheromone-guided “Smart” antimicrobial peptide. Antimicrob. Agents Chemother..

[118] Sullivan R., Santarpia P., Lavender S., Gittins E., Liu Z., Anderson M.H., He J., Shi W., Eckert R. (2011). Clinical efficacy of a specifically targeted antimicrobial peptide mouth rinse: Targeted elimination of *Streptococcus mutans* and prevention of demineralization. Caries Res..

[119] Eckert R., Qi F., Yarbrough D.K., He J., Anderson M.H., Shi W. (2006). Adding selectivity to antimicrobial peptides: Rational design of a multidomain peptide against *Pseudomonas* spp.. Antimicrob. Agents Chemother..

[120] He J., Anderson M.H., Shi W., Eckert R. (2009). Design and activity of a ‘dual-targeted’ antimicrobial peptide. Int. J. Antimicrob. Agents.

[121] Zuckermann R.N., Kodadek T. (2009). Peptoids as potential therapeutics. Curr. Opin. Mol. Ther..

[122] Miller S.M., Simon R.J., Ng S., Zuckermann R.N., kerr J.M., Moos W.H. (1995). Comparison of the proteolytic susceptibilities of homologous L-Amino Acid, D-Amino Acid, and N-Substituted glycine peptide and peptoid oligomers. Drug Develop. Res..

[123] Pompilio A., Crocetta V., De Nicola S., Verginelli F., Fiscarelli E., Di Bonaventura G. (2015). Cooperative pathogenicity in cystic fibrosis: *Stenotrophomonas maltophilia* modulates *Pseudomonas aeruginosa* virulence in mixed biofilm. Front. Microbiol..

[124] Malhotra S., Limoli D.H., English A.E., Parsek M.R., Wozniak D.J. (2018). Mixed communities of mucoid and non-mucoid *Pseudomonas aeruginosa* exhibit enhanced resistance to host antimicrobials. mBio.

[125] Billings N., Millan M., Caldara M., Rusconi R., Tarasova Y., Stocker R., Ribbeck K. (2013). The extracellular matrix component Psl provides fast-acting antibiotic defense in *Pseudomonas aeruginosa* biofilms. PLoS Pathog..

[126] Gläser R., Becker K., von Eiff C., Meyer-Hoffert U., Harder J. (2014). Decreased susceptibility of *Staphylococcus aureus* small-colony variants toward human antimicrobial peptides. J. Investig. Dermatol..

[127] Steadman R., Heck L.W., Abrahamson D.R., Campa M., Bendinelli M., Friedman H. (1993). The role of proteases in the pathogenesis of *Pseudomonas aeruginosa* infections. Pseudomonas aeruginosa as an Opportunistic Pathogen. Infectious Agents and Pathogenesis.

[128] Kuramitsu H.K. (1998). Proteases of *Porphyromonas gingivalis*: What don’t they do?. Oral Microbiol. Immunol..

[129] Joo H.S., Fu C.I., Otto M. (2016). Bacterial strategies of resistance to antimicrobial peptides. Philos. Trans. R. Soc. Lond. B Biol. Sci..

[130] Kang S.J., Park S.J., Mishig-Ochir T., Lee B.J. (2014). Antimicrobial peptides: Therapeutic potentials. Expert Rev. Anti Infect. Ther..

[131] Rončević T., Puizina J., Tossi A. (2019). Antimicrobial peptides as anti-infective agents in pre-post-antibiotic era?. Int. J. Mol. Sci..

[132] Browne K., Chakraborty S., Chen R., Willcox M.D., Black D.S., Walsh W.R., Kumar N. (2020). A new era of antibiotics: The clinical potential of antimicrobial peptides. Int. J. Mol. Sci..

